# Functional Analysis of the Engineered Cardiac Tissue Grown on Recombinant Spidroin Fiber Meshes

**DOI:** 10.1371/journal.pone.0121155

**Published:** 2015-03-23

**Authors:** Alexander Teplenin, Anna Krasheninnikova, Nadezhda Agladze, Konstantin Sidoruk, Olga Agapova, Igor Agapov, Vladimir Bogush, Konstantin Agladze

**Affiliations:** 1 Moscow Institute of Physics and Technology, Institutski pereulok 9, Dolgoprudny, Moscow region, 141700, Russia; 2 The State Research Institute for Genetics and Selection of Industrial Microorganisms, 1st Dorozhny proezd 1, Moscow 117545, Russia; 3 The Shumakov Research Center for Transplantology and Artificial Organs, Shchukinskaya 1, Moscow, 123182, Russia; Centro Cardiologico Monzino, ITALY

## Abstract

In the present study, we examined the ability of the recombinant spidroin to serve as a substrate for the cardiac tissue engineering. For this purpose, isolated neonatal rat cardiomyocytes were seeded on the electrospun spidroin fiber matrices and cultured to form the confluent cardiac monolayers. Besides the adhesion assay and immunostaining analysis, we tested the ability of the cultured cardiomyocytes to form a functional cardiac syncytium by studying excitation propagation in the cultured tissue with the aid of optical mapping. It was demonstrated that recombinant spidroin fiber meshes are directly suitable for the adherence and growth of the cardiomyocytes without additional coating with the attachment factors, such as fibronectin.

## Introduction

A rapid development in the field of tissue engineering in the last decade has created an increased demand for new prospective materials that are able to serve as scaffolds [1–5]. The new materials are especially important in particular for the cardiac tissue engineering because of the multifaceted requirements for the cultured cardiac tissue constructs. So far, no ideal or most appropriate material has been chosen since in addition to the common bio-scaffold properties such as biocompatibility (non-toxic), and safe degradation in the body, cardiac tissue scaffolds must provide two major properties: 1) mechanical and elastic characteristics matching to the host cardiac tissue, and 2) the environment for functional electrophysiological unity of the cardiac cells.

In the present study, we examined cardiac tissue culture grown on the recombinant spidroin meshes. Recombinant analogs of spidroins (dragline silk proteins of spider web) represent a class of the promising materials for the tissue engineering. They incorporate great cell adhesion properties, excellent mechanical strength, and impressive elasticity. Recombinant spidroin materials were successfully used for designing bone, tendon and cartilage implants [6–8], for controlled drug delivery [9, 10], wound dressing[11] and tissue engineering[12–14]. An important advantage of recombinant spidroins over natural silk, containing RGD motive, is the ability to include various functional motive sequences. For instance, recently the possible role of a combination of two alginate-attached peptides, the adhesion peptide G_4_RGDY and heparin-binding peptide G_4_SPPRRARVTY (HBP) peptide, in cardiac tissue regeneration was shown [15].

The genes encoding two recombinant analogs of natural dragline silk proteins, spidroins 1 and 2, proteins rS1/9 (old/other designation—1F9) [16] and rS2/12 (old/other designation—2E12) [17] were designed, synthesized and subcloned into the yeast cells *Saccharomyces cerevisiae* under the control of the GAL1 promoter. rS1/9 is an analog of natural spidroin 1, one of the two proteins of dragline silk of the orb weaver spider *Nephila clavipes*, and characterized by molecular weight (MW) of 94 kDa. rS2/12 is an dragline silk spidroin 2 analog of the orb weaver spider *Nephila madagascariensis*, and has MW of 113 kDa. They show impressive biocompatibility and ability to accelerate tissue regeneration, participating in the vascularization and nerve formation in the wound area in animal models. Moreover, they demonstrate slow biodegradability, low immunogenicity and ability to spontaneous micro- and nanoporosity [14, 18]. The RGD sequence is the most often employed peptide to stimulate cell adhesion and proliferation on different biomaterials, as it is a ligand of the cell adhesion integrin receptor family. RGD-peptides that interact with integrins on the cell surface (including cardiac cells), initiate a cascade of reactions in the cell that affect many aspects of cellular behavior [19, 20].

While the outstanding mechano-elastic features of the silk fibers as well as the potential power of the genetic manipulations with silk proteins are well documented [4, 21, 22], especially, in the works of Kundu, *et al*. [23–25], the physiological characteristics of the silk-based engineered excitable cardiac tissue are not yet fully studied. For example, there is an apparent lack of knowledge of electrophysiological characteristics of the cardiac tissue grown on the silk proteins. Thus, we focused on the excitable features of the grown cardiac tissue layers: the main goal of the study was to prove, that besides only survival and growth, the engineered cardiac tissue is able to transmit organized electrical signals for the orchestrated contracting, potentially taking part in the crucial heart’s job: pumping blood.

For this purpose, isolated neonatal rat cardiomyocytes were seeded on the fiber matrices and cultured to form confluent cardiac monolayers. Cardiac cells successfully adhered and grew on the spidroin fiber meshes obtained by electrospinning, forming contractile and excitable cell network supported by the spidroin fibers. We tested adherence of the cardiac cells to the different types of recombinant spidroins, containing and non-containing RGDS motif. The excitability and conduction of the excitation waves in the grown cardiomyocyte layers were analyzed with the aid of optical mapping [3, 26], i.e., recording of the excitation waves with fluorescent markers.

## Materials and Methods

### 1. Preparation of electrospun spidroin substrate

#### 1.1. Materials

The genes encoding two recombinant analogs of spidroins—proteins rS1/9 (old/other designation—1F9) [16] and rS2/12 (old/other designation—2E12) [17] were subcloned into the yeast *Saccharomyces cerevisiae* by integration into chromosome under the control of the GAL1 promoter using a repliconless expression vector.

**Fig 1:**
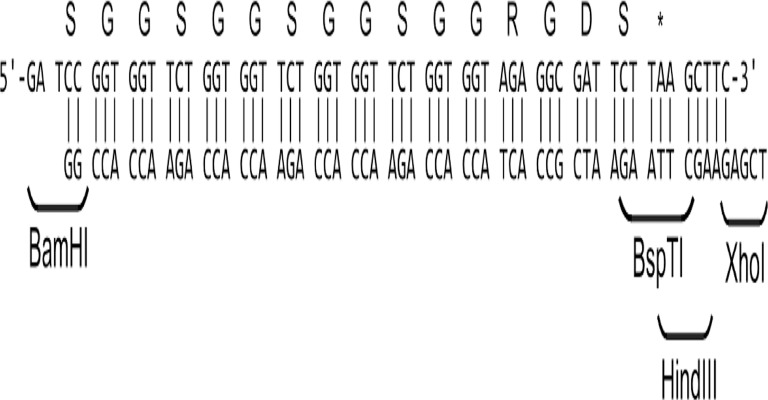
Gene construction for Linker-RGDS.

RGDS fragment was attached to the C-end of rS2/12 protein through linker (GGS)_3_GG using standard protocols of molecular cloning. (GGS)_3_GG is an artificial linker between the protein molecule and the sequence RGDS, the presence of which can increase the likelihood of exposure of this sequence on the surface of the protein substrate. For this purpose, two mutually complementary oligonucleotides were synthesized and annealed to each other (Fig. 1).

Double stranded fragments with sticky ends, corresponding to the sticky ends of BamHI and XhoI restriction sites were subcloned into the previously obtained bireplicon plasmid carrying rs2/12 gene at the BamHI and XhoI restriction sites at the 3'-end of the gene.

The resulting plasmid was transformed into yeast cell *Saccharomices cerevisiae*. As a result, a yeast–producer of modified protein rS2/12-(GGS)3GG-RGDS was obtained. The expressed rS1/9 and rS2/12-(GGS)3GG-RGDS (rS2/12-Linker-RGDS) proteins were purified from water insoluble fraction of yeast cells by the previously developed method, including chromatography on a strong cation exchanger SP Sepharose Fast Flow (Amersham Biosciences, Uppsala, Sweden) in a fast protein liquid chromatography system by a two-step pH-gradient elution [27]. A high purity of the samples was confirmed by sodium dodecyl sulfate electrophoresis. The purified proteins were dialyzed against deionized water, lyophilized, and stored at +4°C until use. Natural silk from *Bombyx mori* cocoons was boiled for 1 h in a 0.03 M solution of NaHCO_3_, with following thorough rinsing in distilled water to remove the glue-like sericin proteins and wax.

#### 1.2. Electrospining of PCL and spidroin nanofibers

Recombinant spidroins, as well as a *Bombyx mori* fibroins were dissolved in the hexfluorisopropanol at concentrations of 15–25%. In the case of [rS1/9 + rS2/12-Linker-RGD] mixture, ingredients were taken at a 1:1 ratio for both pairs.

The polycaprolacton (PCL) solution was prepared by dissolving it in hexfluorisopropanol at concentrations of 10–15%. The prepared PCL, spidroin and fibroin solutions were electrospun using Nanon-01 electrospinning setup (MECC CO.,LTD). All these solutions were loaded into the 2 ml syringe and ejected through the 20 gauge blunt tip needle at a flow rate of 0.1–1ml/h. The applied voltage between the syringe tip and the grounded collector was in the range from 5 to 10 kV. Nanofibers were electrospun directly onto the surface of either 12mm or 22mm diameter cover glass deposited on the grounded collector. After completing the electrospinning process, the spidroin fibrous substrates were immersed in 96% ethanol for 24 hours in order to initiate alignment of the β-sheets in the silk fibers, to give strength and water resistance. Then the spidroin substrates were washed in PBS for 24 hours and dried afterwards. The non-adhesive to live cells pure PCL nanofibers substrates and several specimens of *Bombyx mori* fibroin mats were coated with a solution of human plasma fibronectin (0.16 mg / ml in PBS, Gibco, USA) to produce a cell adhesive matrix.

### 2. Cardiac cell isolation, seeding, cultivation

All studies conformed to the Guide for the Care and Use of Laboratory Animals, published by the United States National Institutes of Health (Publication No. 85–23, revised 1996) and approved by the Moscow Institute of Physics and Technology Life Science Center Provisional Animal Care and Research Procedures Committee, Protocol #A2–2012–09–02. Cardiac cells were isolated from the ventricles of 1–3 day old neonatal Wistar rats, according to the Worthington protocol (http://www.worthingtonbiochem.com/NCIS/default.html). Then isolated cells were seeded on the specimens at 1.5–3 ×10 cells/cm cell density and cultivated in DMEM culture medium. After 3–5 days of cultivation primary culture cells formed monolayers and performed coordinated contraction activity.

#### 2.1. Sample fabrication for the functional analysis of spidroin fibrous substrates

In order to directly compare functional properties of the cardiac monolayers grown on different spidroin mesh types to the monolayers grown on the fibronectin coated surface, composite samples were fabricated. For this purpose only half of the cover glass was covered by the polymer fibers and the remaining area was coated by the fibronectin. Then cells were seeded on the specimens as described above.

### 3. Cardiac cell adhesion assay

To analyze cell adhesion to the variety of different surfaces (fibronectin covered glass and fibronectin coated PCL fibers, fibroin fibers and fibroin fibers coated by fibronectin, rS1/9, rS2/12-Linker-RGDS, rS1/9+rS2/12-Linker-RGDS [1:1 mixture] recombinant spidroin meshes of various fiber diameters) the substrate specimens were placed into 24-well plate. Then isolated cardiac cells at the concentration of 1.5 ×10 cells/cm were seeded on them. After 12 hours cells were washed in PBS for 10 minutes by shaking on nutator, then fixed in 4% paraformaldehyde and stained by Alexa Fluor 488 Phaloidinin Conjugate for actin filaments visualization and DAPI for outlining of nuclei according to protocol described in [3]. Images of the stained specimens were captured with IX-71 inverted fluorescent microscope and DP72 Olympus camera. Then the cells were counted using ImageJ software and comparative analysis of the adhesion properties of different substrates was performed.

### 4. Scanning electron microscopy

In preparation for the SEM imaging all specimens were covered with 10nm layer of gold utilizing 150R/ES spatter coater (Quorum Technologies, UK). JEOL 6510-A was used for characterization of the shape and diameter of nanofibers. Accelerating voltage was 10kV. ImageJ software and Wolfram Mathematica were used for the automated measurement of fiber diameter at each acquired photograph. Average fiber size was calculated according to the size distribution histogram.

### 5. Fluorescent cardio specific markers labeling and imaging

Immunofluorescent staining of cardiomyocytes monolayers on rS2/12-linker-RGDS recombinant spidroin substrates was performed by using secondary and primary antibodies according to the previously described protocol [28]. In our work we used anti-α-actinin, anti-connexin43 primary antibodies for the cardiomyocytes specific labeling, Alexa Fluor-488 Phaloidinin conjugate for labeling α-actin skeleton of the cell, and DAPI for labeling nuclei of the cells.

### 6. Optical mapping of excitation waves

For monitoring activity and excitation waves recording, the 4–5 days old monolayer cultures were loaded with the Ca^2+-^sensitive indicator Fluo-4-AM (Invitrogen, USA). After staining, the medium was exchanged with Tyrode’s solution (Sigma—Aldrich Co., USA) and kept at room temperature during the observations [3]. The excitation waves were monitored using a high-speed imaging setup (Olympus MVX-10 Macro-View fluorescent microscope equipped with high-speed Andor EM-CCD Camera 897-U at 68 fps).

For the initiation of the excitation waves, cathodal point stimuli were applied at various frequencies and at different sites of the sample using 500 μm diameter platinum wire and grounded looped platinum wire return electrode placed along the dish wall about 10 mm away from the cover slip.

All videos were processed in ImageJ software and Wolfram Mathematica using custom-made temporal 8-term Daubechie 3 iteration wavelet shrinkage filter and Gaussian blurring for spatial denoising (NIH, Maryland, USA, http://rsb.info.nih.gov/ij). Pseudocardiograms of the composite samples covered in half by spidroin and in half by the fibronectin were plotted in order to compare the frequency response to the external point stimuli. The conduction velocity calculation was performed on each half of the composite sample by building time-space plots as described in [3].

## Results

### 1. Manufacturing and morphological characterization of the electrospun spidroin fibers

**Fig 2:**
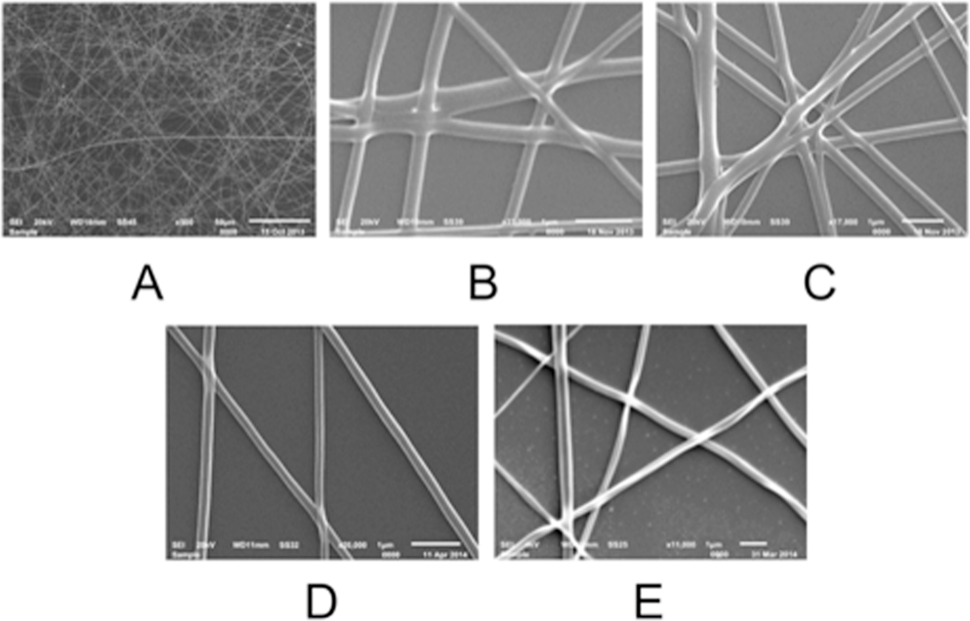
SEM micrographs of recombinant spidroin and fibroin *Bombyx mori* fibrous substrates. (a) low magnification of rS1/9 spidroin substrate (frame size 257 μm × 192 μm), (b) high magnification micrograph of rS1/9 spidroin fibers (frame size 5.6 μm × 4.2 μm), (c) high magnification micrograph of rS2/12-Linker-RGDS spidroin fibers (frame size 7.7 μm × 5.8 μm), (d) high magnification micrograph of rS1/9+rS2/12-Linker-RGDS spidroin (1:1) mixture fibers (frame size 6.3 μm × 4.7 μm), (e) high magnification micrograph of fibroin *Bombyx mori* fibers (frame size 11.4 μm × 8.5 μm).

The polymer fibers were electrospun as described in Materials and Methods. The electrospinning parameters and conditions were optimized for the formation of bead-free smooth and uniform nanofibers. The fibers of different diameters were produced by varying the distance between the tip and collector and adjusting the concentration of the polymer solution. The deposited fibers were distributed in random directions, and the average pore size for all fibrous scaffolds was around 2 μm. The SEM microphotographs (Fig. 2) show the examples of nanofiber matrices. The same density samples were used for the seeding cells. We should also note that: 1) the polymer density was controlled over relatively large areas (c.a. 5 mm × 5 mm), while it could vary in the microscopic regions; 2) we did not notice any change in the adhesion if the average pore size in the fiber mesh was lower than the average cell size (c.a. < 10 mμ).

### 2. Cardiac cells growth on the substrate covered with spidroin nanofibers

#### 2.1. Adhesion assay

The first and imperative stage of the cardiomyocyte culture development is attachment of cells to the substrate. The adherent cells have a chance for further development and formation of a functional syncytium, while non-adherent cells die. In order to compare the adhesive properties of the different spidroin substrate variations between themselves and to the common fibronectin coated glass, fibronectin coated PCL fibers and widely used *Bombyx mori* fibroin substrates, the adhesion assay was developed (1.3).

**Fig 3:**
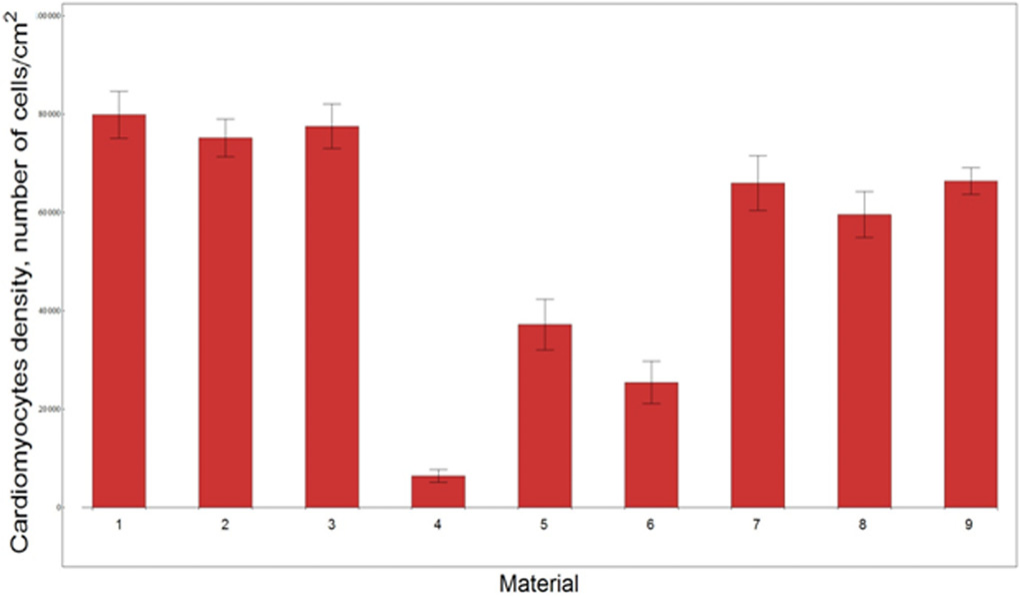
Quantitative analysis of an adhesion assay of fibronectin-coated common substrates and silk polymer substrates. 1-fibronectin, 2- PCL coated with fibronectin (thickness 0.3 μm), 3- fibroin Bombyx mori coated with fibronectin (thickness 0.25μm), 4-fibroin Bombyx mori (thickness 0.25 μm), 5- rS1/9 (thickness 0.4 μm), 6- rS2/12 (thickness 0.4 μm), 7- rS2/12-Linker-RGDS(thickness 0.35 μm), 8- rS2/12-Linker-RGDS (thickness 0.25 μm), 9- rS1/9+rS2/12-Linker-RGDS 1:1(thickness 0.6μm)

The results of the adhesion testing for the various substrates are presented in Fig. 3. Each bar in the histogram represents an average cell density (i.e. number of cells per cm^2^) of adherent cardiomyocytes for the corresponding substrate type. A total of 24 samples were tested for each type of substrate, and the average of the five trials was used.

The histograms show that the diameter of the fibers does not significantly affect the amount of adherent cardiac cells. The number of cardiomyocytes adhered to rS2/12-Linker-RGDS, rS1/9+rS2/12-Linker-RGDS (1:1 mixture) recombinant spidroins meshes containing RGDS motif as well as to rS2/12 and rS1/9 recombinant spidorin mesh non-containing RGDS motif were approximately 10%, 60% and 50% less than the number of cardiomyocytes adhered to the fibronectin coated substrates, respectively. The amount of attached cardiac cells to the plain *Bombyx mori* fibroin substrates was 90% less than the number of cardiac cells attached to fibronectin coated surfaces. The number of adhering cells to all fibronectin covered surfaces did not vary significantly between fibroin coated PCL meshes, fibroin coated *Bombyx mori* meshes and fibroin coated glass coverslips.

#### 2.2. Morphological characterization of the cardiac monolayer grown on spidroin fibrous substrate

**Fig 4:**
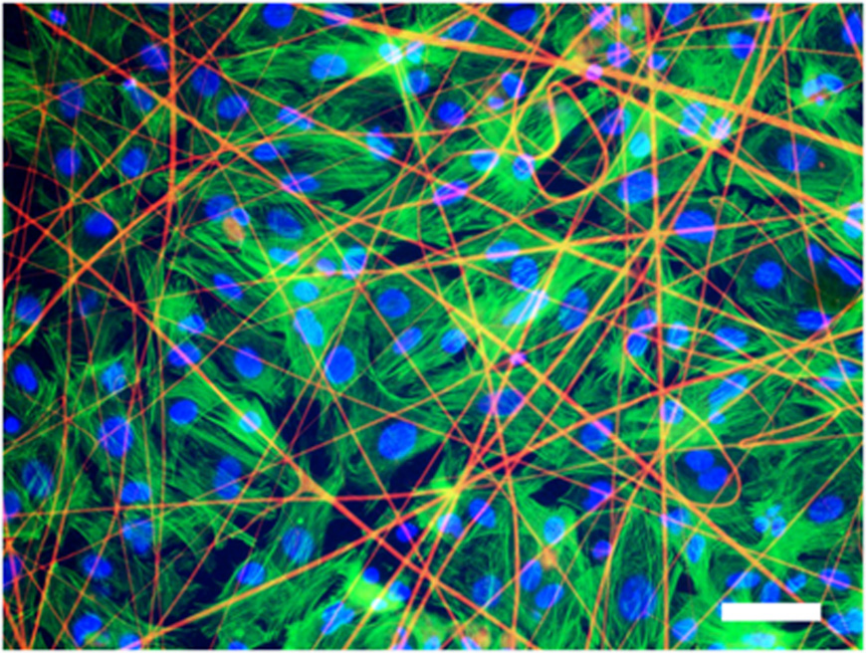
Fluorescent image of α-actin (green) stained with Alexa Fluor-488 Phaloidinin conjugate and nuclei (blue) stained with DAPI of cultured rat cardiomyocytes monolayers grown on fibrous rS2/12-Linker-RGDS spidroin substrate (fibers are yellow). Scale bar—50 μm.

**Fig 5:**
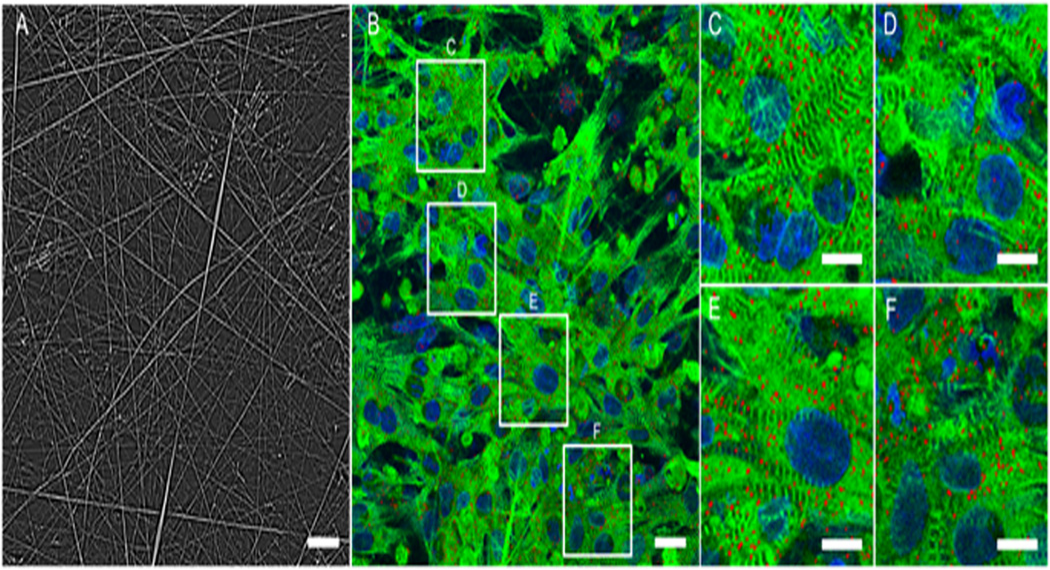
Immunostaining of cardiomyocytes monolayers grown on fibrous rS2/12-Linker-RGDS spidroin substrate. A: Photo of fibrous substrate, scale bar 20 μm.B: α-actinin (green), connexin (red), nuclei (blue) labeling, scale bar 20 μ.m (C-F) Close-ups of segments on image B, scale bar—5 μm.

After 3–5 days of cultivation on rS1/9, rS2/12, rS2/12-Linker-RGDS and rS1/9+rS2/12-Linker-RGDS (1:1 mixture) recombinant spidroin meshes, cardiomyocytes formed the confluent monolayers exhibiting spatially coherent contractile activity. The obtained confluent monolayers were stained with primary α-actinin, Cx43 connexin antibodies and suitable secondary antibodies, Phaloidinin conjugated dye for α-actin labeling, and DAPI. The stained samples were analyzed using laser scanning confocal microscope Zeiss LSM-710. The examples of acquired fluorescent images are shown in Fig. 4 and Fig. 5. The figures show cardiac monolayers only for the rS1/12-Linker-RGDS fibrous mat. The pictures obtained for other recombinant spidroin substrates didn’t differ significantly. As represented in Fig. 5, cardiac monolayer cells expressed cardio-specific α-actinin proteins which were localized to the Z-disks of the cardiac cell, as well as cardiac specific Cx43 connexin, and cytoskeletal α-actin. This immunostaining indicates that the majority of monolayer’s cells are cardiomyocytes.

### 3. Functional characterization of the cardiac monolayers grown on fibrous spidroin mesh substrate

The main function of the cardiac tissue is pumping blood through the coordinated contraction of muscle fibers. The coordination is accomplished by the propagating excitation waves triggering contraction on the cell level. Thus, for engineered cardiac tissue both processes, contraction and propagation of excitation are equally important and both were verified in our experiments.

#### 3.1. Contractile activity

The 3–5 days old cultures were observed with the aid of phase-contrast microscope and confirmed as the confluent cardiac monolayers. At this stage of development, they usually exhibited spontaneous contractile activity, which served as the first indication of the grown unified cardiac tissue.

**Fig 6:**
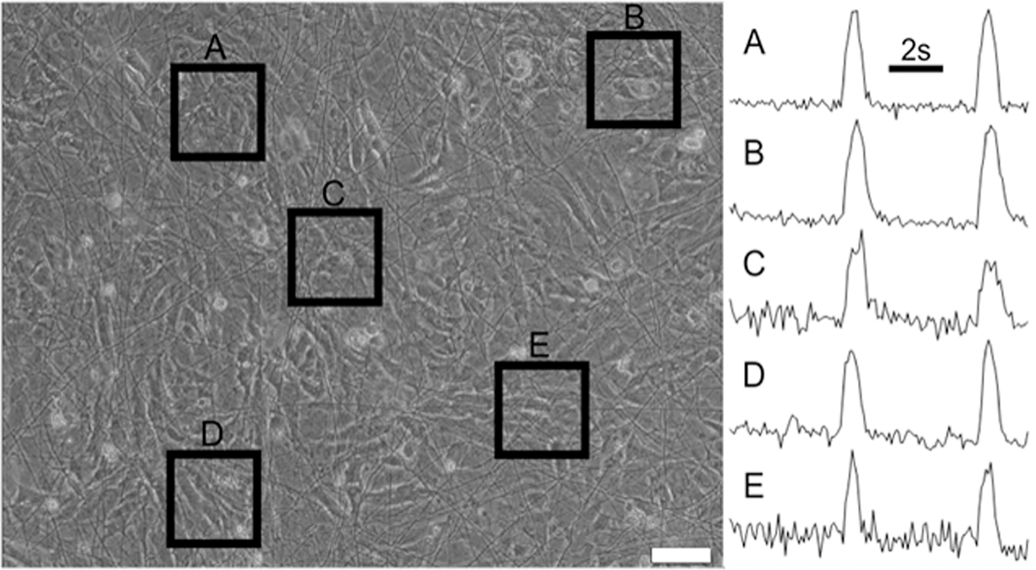
Representation of coordinated contractile activity of cardiomyocytes monolayer grown on rS2/12-Linker-RGDS spidroin fibrous substrate. Video freeze-frame of contractile activity, scale bar 20 μm. (A-E): Graphs of intensity variation over time on corresponding outlined regions of interest.

The recorded videos of synchronous contraction in the cardiac cell monolayer grown on rS2/12-Linker-RGDS fibrous mesh are shown in S1 Movie and S2 Movie, the similar synchronous contractions were observed in the cardiac cultures grown on rS1/9, rS2/12 and rS1/9+rS2/12-Linker-RGDS(1:1 mixture) fibrous substrates (the data not shown). Analysis of S2 Movie is presented in Fig. 6. In a course of contraction, intensity of pixels in the video changes with time, and the intensity values are plotted over time for various sites of the sample, (Fig. 6). The recorded peaks of intensity correspond to the moment of cells contraction. Subsequent processing of the plots obtained demonstrates that the observed peaks are simultaneous for the different sites of the sample and thus reveals the correlation of the spontaneous contractions.

#### 3.2. Excitation wave propagation on spidroin fibrous mesh substrates

**Fig 7:**
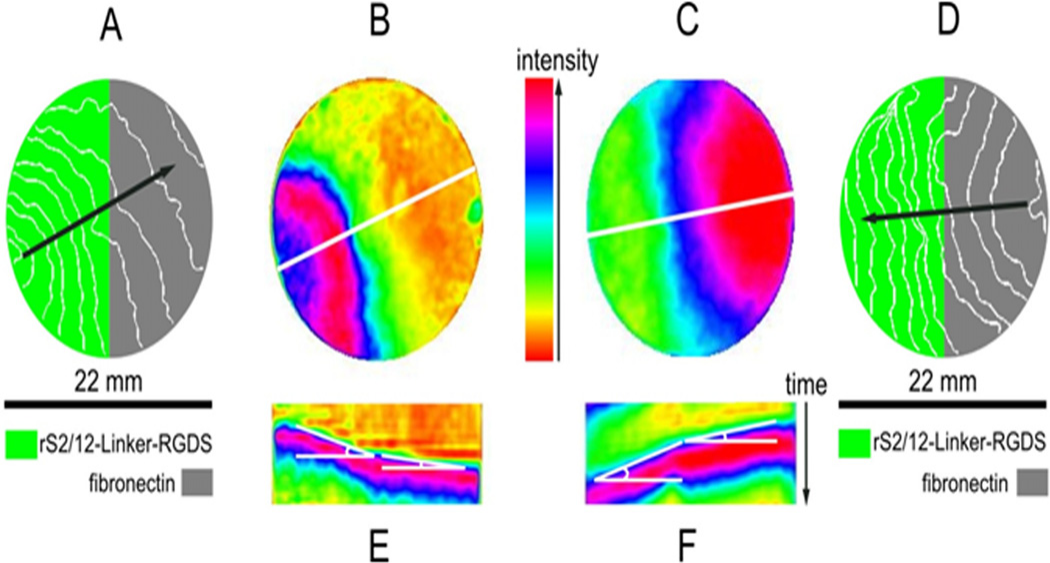
Verification of suitability of monolayers grown on rS2/12-Linker-RGDS spidroin fibrous substrate for excitation wave propagation. A, D: isochrone maps for point stimulation from rS2/12-Linker-RGDS fibers and fibronectin coated side, respectively. Line interval: 60 ms. Arrows show direction of wave propagation in each case. B, C—freezed frames of processed optical mapping videos represented by A,D pictures, correspondingly. E, F—time-space plots for white lines shown on pictures B, C with marked angles for velocity calculation.

The samples of the cardiac tissue were prepared as described in 1.2.1.and the excitation waves monitored and recorded as described in 1.6. At least 10 samples for each type of substrate material were analyzed. During video capturing, a sequence of external voltage point stimuli was applied, and the excitation waves propagated through both recombinant spidroin and fibronectin sides of the sample. Thus, it was possible to directly compare conduction speed in the cardiac tissue grown on different substrates, in pairs. A typical example of the excitation propagation in this experiment is shown in Fig. 7 and also can be viewed in S3 Movie and S4 Movie. These videos were used for building time-space plots along the white lines shown in the panels B and C, (Fig. 7) and are represented on the panels E and F, respectively. The propagation speed can be measured by calculation of the tangent value of angles, which are marked by white lines in the panels E and F (Fig. 7). In this experiment directly measured conduction speed on fiber mesh consisting of rS2/12-Linker-RGDS recombinant spidroin, appeared about two times lower than the conduction speed on fibronectin coated glass. The conduction speeds also can be compared using the isochrones maps shown in panels A and D, (Fig. 7), where on the recombinant spidroin side of the sample (green) isochrones are visibly denser spaced than on the fibronectin side (grey). The conduction speed ratio for cultures grown on different substrates and the conduction speed on fibronectin coated glass is shown in Table 1.

**Table 1:** Comparison of functional properties of cardiac monolayers on different substrates.

Material/Properties	Velocities ratio	Critical period of stimulation, ms
PCL	1	350±25
rS1/9	0.57±0.08	500±25
rS2/12-linker-RGDS	0.44±0.05	400±25
rS1/9+rS2/12-Linker-RGDS	0.44±0.05	500±25

**Fig 8:**
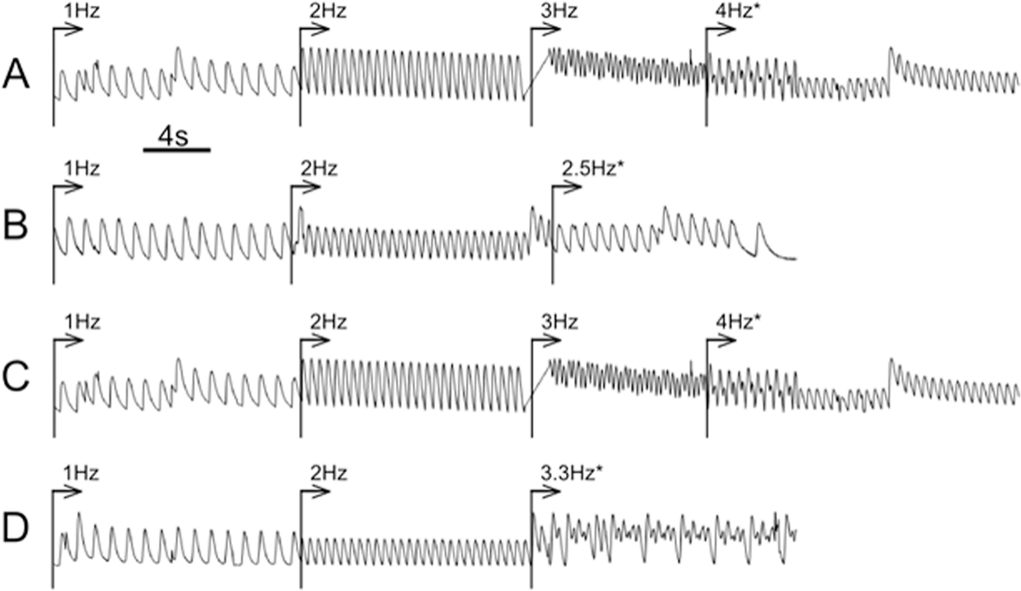
Pseudocardiograms of wave propagation on various types of substrates. Arrows depict starting moments of an electrical stimulation. Decreased frequency or transformation rhythm responses are marked by the asterisks. A—PCL+fibronectin pseudocardiogram for fibronectin coated PCL fibrous substrate. B—pseudocardiogram for rS1/9 fibrous substrate. C—pseudocardiogram for rS2/12-Linker-RGDS fibrous substrate. D—pseudocardiogram for rS1/9+rS2/12-Linker-RGDS fibrous substrate. The calculated critical frequencies for various substrates are presented in Table 1.

Besides the conduction speed, an important characteristic of the cardiac tissue is the ability to capture the rhythmic excitation supplied by natural or external pacemakers. The instabilities occurring at a higher stimulation rate, create prerequisites for lethally dangerous tachyarrhythmia. Thus, we compared the ability of cardiac tissue grown on different substrates to capture the external rhythm using the composite samples similar to those used to measure conduction velocity in pairs. External pacing was applied to both parts of the sample and the highest frequency at which one-to-one response to external stimuli occurs was determined. Higher pacing rates usually caused so-called rhythm transform, i.e. skipping one or more beat, and decreased the response frequency. The critical capturing frequency was determined for each substrate material from pseudocardiograms, examples of which are presented in Fig. 8. The pseudo-cardiograms were built by monitoring the fluorescence intensity in a small area (about 2 mm × 2 mm) of the sample, covered by the cardiac cells on the corresponding substrate.

## Discussion

Silk proteins represent a very promising class of tissue engineering materials due to a combination of several unique features, such as:

- Exceptional elasticity
- Biocompatibility (it is fully “organic” product)
- Availability for genetic modifications
- Suitability for the electrospinning process

Previously, promising results were obtained by a group of researchers using silk protein fibroin from Indian tropical tasar *Antheraea mylitta* as a scaffold for engineering a cardiac patch in vitro [24, 29]. They tested cell adhesion, cellular metabolic activity, and response to extracellular stimuli of the cultured tissue. Also, it was shown that implantation of the chitosan-hyaluronan/silk fibroin (chitosan-HYA/SF) cardiac patches to the infarcted rat heart improved their survival rate, thus showing excellent biocompatibility of silk polymers *in vivo* [30]. In our experiments, besides adhesion assay and immunostaining analysis, we focused on the ability of the cultured cardiomyocytes to form a functional cardiac syncytium by studying excitation propagation in the cultured tissue.

The present study had been devoted to testing the appropriateness of the recombinant analogs of spider proteins, spidroins, to the cardiac tissue engineering. The study aimed to answer the following questions:

1) Do the cardiac cells attach to the recombinant spidroin substrates, and what is the need in the additional attachment factors?
2) Do the cardiac cells survive, grow and form confluent layers?
3) Do the grown tissue layers form a cardiac syncytium and are they fully functional, i.e. capable of coordinated contraction?

We may say “yes” to all questions. Although, as shown in our studies, the number of cardiomyocytes primarily adhered to all tested recombinant rS2/12-Linker-RGDS spidroin containing meshes, rS2/12 and rS1/9 fibrous meshes was about 10%, 60% and 50% lower than the number of cardiomyocytes attached to the fibronectin coated glass and PCL substrates, this had no effect on the ability of cells to grow and develop into confluent cell sheets. No additional attachment factors were needed (i.e. special type of coating). The presence of RGDS motif only affected adherence properties of matrices, while functional and morphological properties of the resulting cell monolayers grown on all types of recombinant spidroin substrates were almost equal after 3–5 day of cultivation.

As for the inspection of the grown tissue, periodic contraction of cardiomyocyte layers could be easily observed by visual (microscopic) examination and, in fact, serves as the most common and readily available indicator of the functionality of the grown tissue. We put this simple analysis a little further, proving the coherence of the spontaneous contractions throughout the different sites of the tissue sheet.

Finally, the optical mapping of excitation proved that cells indeed form the syncytium, and the excitation pulse travels through the grown tissue and controls the cell functioning as in the cardiac tissue *in vivo*. The observed conduction speed for the cells grown on recombinant spidroin substrates was about 50% of its value for the PCL substrates coated with fibronectin. However, in these model experiments, it is hard to tell what values would fit better for the real heart tissue *in vivo*. With a view of this, we are planning further study involving measuring of the expression levels of gap-junction proteins and voltage-gated ion channels, as well as of their activity depending on the substrate material on which tissue is grown.

## Conclusions

We have demonstrated that recombinant spidroins can be used to produce electrospun fiber mesh substrates. These substrates are suitable directly for the cardiomyocytes attachment and growth without additional coating with the attachment factors, such as fibronectin.

## Supporting Information

S1 Movie The recorded video of synchronous contraction in the cardiac cell monolayer grown on rS2/12-Linker-RGDS fibrous mesh. (AVI)

S2 Movie The recorded video of synchronous contraction in the cardiac cell monolayer grown on rS2/12-Linker-RGDS fibrous mesh. (AVI)

S3 Movie The example of excitation propagation in the experiment shown in Fig. 7 from right to left. (AVI)

S4 Movie The example of excitation propagation in the experiment shown in Fig. 7 from left to right. (AVI)
